# The Wandering Pancreas: A Rare Case of Complex Hiatal Hernia Presenting With Thoracic Symptoms

**DOI:** 10.7759/cureus.105272

**Published:** 2026-03-15

**Authors:** Almahdi El Hassani, Hiba Abbay, Badr Slioui, Abdelilah Mouhsine, Salah Bellasri

**Affiliations:** 1 Department of Radiology, Mohammed VI Hospital/Cadi Ayyad University, Marrakech, MAR; 2 Department of Radiology, Avicenne Military Hospital, Faculty of Medicine and Pharmacy, Cadi Ayyad University, Marrakech, MAR

**Keywords:** collar sign, hiatal hernia, mdct, pancreatic herniation, posterior mediastinum, type iv hernia, vascular looping

## Abstract

Hiatal hernia (HH) is defined as the protrusion of abdominal contents through the esophageal hiatus of the diaphragm into the mediastinum. While type I sliding hernias are common, type IV hernias, which are characterized by the herniation of organs other than the stomach, such as the colon or spleen, are uncommon. Even more exceptional is the herniation of the pancreas, given its retroperitoneal fixation. We report the case of a 60-year-old female with a history of obesity (BMI 32 kg/m²) who presented with a six-month history of chronic dry cough, dyspnea, and retrosternal heaviness. Initial chest radiography revealed a retrocardiac opacity with an air-fluid level that mimicked a mediastinal mass. Urgent multidetector CT (MDCT) showed a giant type IV HH (defect size 5.5 cm) containing the stomach, transverse colon, and notably, the body and tail of the pancreas. The diagnosis was confirmed by identifying the characteristic "collar sign" and "vascular looping" of the splenic vessels and left gastric artery extending into the thorax. Although the patient did not display signs of acute pancreatitis or obstruction, the large defect size and vascular involvement necessitated a surgical referral to prevent future incarceration. This report highlights the critical role of MDCT in identifying the "vascular loop" sign, which helps distinguish pancreatic herniation from mediastinal neoplasms and avoid iatrogenic injury during surgical repair.

## Introduction

Hiatal hernia (HH) is defined as the protrusion of abdominal contents through the esophageal hiatus of the diaphragm into the mediastinum. Epidemiologically, HH is highly prevalent, and major risk factors for its development include advanced age and high BMI. Research indicates that patients with a BMI >30 kg/m² have a four- to fivefold higher risk of developing HH compared to those with a normal BMI [1]. Pathophysiologically, HH arises due to progressive laxity of the phrenoesophageal ligament and widening of the diaphragmatic hiatus, a process that is worsened by chronically elevated intra-abdominal pressure [2].

Hiatal hernias are classified anatomically into four types [3,4]. Type I sliding hernias are the most common, representing more than 90% of cases, and occur when the gastroesophageal (GE) junction and gastric cardia move axially above the diaphragm. Type II pure paraesophageal hernias occur when the GE junction remains in its normal anatomical position below the diaphragm, while the gastric fundus herniates alongside the esophagus. Type III mixed hernias combine elements of both, with both the GE junction and gastric fundus protruding into the mediastinum. Finally, Type IV hernias, also known as giant or complex paraesophageal hernias, are characterized by a large diaphragmatic defect that allows abdominal organs other than the stomach, such as the colon, spleen, pancreas, or small bowel, to herniate into the sac [5,6].

Clinical features can range from asymptomatic presentations or mild gastroesophageal reflux disease (GERD) in type I hernias to mechanical, mass-effect symptoms, such as dysphagia, early satiety, postprandial dyspnea, and retrosternal heaviness, seen in giant type IV hernias [3]. Consequently, the differential diagnosis for these thoracic symptoms is broad and requires clinicians to exclude acute cardiopulmonary conditions, primary esophageal motility disorders, and posterior mediastinal masses, including neurogenic tumors or bronchogenic cysts. Diagnostic tests typically involve barium esophagram and endoscopy; however, contrast-enhanced multidetector CT (MDCT) remains the gold standard for complex type IV hernias, as it accurately delineates the herniated anatomy and identifies acute complications [3].

The management of HH depends on the hernia type and the presence of complications [4]. Type I hernia is mainly treated with medical therapy, including proton pump inhibitors and lifestyle modifications. In contrast, types II-IV pose significant risks for serious mechanical complications, such as gastric volvulus, incarceration, strangulation, bowel ischemia, and Cameron lesions (gastric mucosal erosions that can cause chronic bleeding). Therefore, symptomatic giant hernias often require elective laparoscopic repair with fundoplication [3,4].

However, herniation of the pancreas is an extremely rare occurrence. Anatomically, the pancreas is a retroperitoneal organ, firmly attached to the posterior abdominal wall and the duodenum via the ligament of Treitz and the transverse mesocolon [7]. This fixation limits its mobility, making intrathoracic displacement highly unusual. The purpose of this report is to present a rare case of a type IV HH involving the pancreas. While prior reports have documented pancreatic herniation, this report specifically emphasizes the "vascular loop" sign of the splenic vessels on MDCT as a definitive teaching point to differentiate this anomaly from mediastinal neoplasms and to guide surgical management, since this entity can often mimic other posterior mediastinal masses.

## Case presentation

A 60-year-old female presented with a six-month history of chronic dry cough, exertional dyspnea, regurgitation, and retrosternal heaviness. Postprandial worsening of her dyspnea suggested a mass-effect mechanism, wherein the distended, herniated stomach compressed adjacent intrathoracic structures. She denied acute chest pain, dysphagia, vomiting, gastrointestinal bleeding, or sleep apnea. Her medical history included hypertension and obesity (BMI of 32 kg/m²). Vital signs were stable: blood pressure was 130/85 mmHg, heart rate was 78 bpm, respiratory rate was 18/min, and oxygen saturation was 98% on room air. Pulmonary auscultation revealed diminished breath sounds at the left lung base. Abdominal examination was soft and non-tender, with no palpable masses or guarding. Routine laboratory investigations (Table 1) were performed to rule out infectious or inflammatory etiologies and were largely unremarkable.

**Table 1:** Patient's laboratory values at initial presentation

Analyte	Patient value	Reference range
Hemoglobin	13.8 g/dL	12.0 - 15.5 g/dL (female)
White blood cells (WBC)	8,700/µL	4,500 - 11,000 /µL
C-reactive protein (CRP)	2.6 mg/L	< 5.0 mg/L
Lipase	45 U/L	13 - 60 U/L
Glucose	1.1 g/L	0.70 - 1.00 g/L
Aspartate aminotransferase (AST)	15 U/L	10 - 40 U/L
Alanine aminotransferase (ALT)	36 U/L	10 - 40 U/L

**Figure 1:**
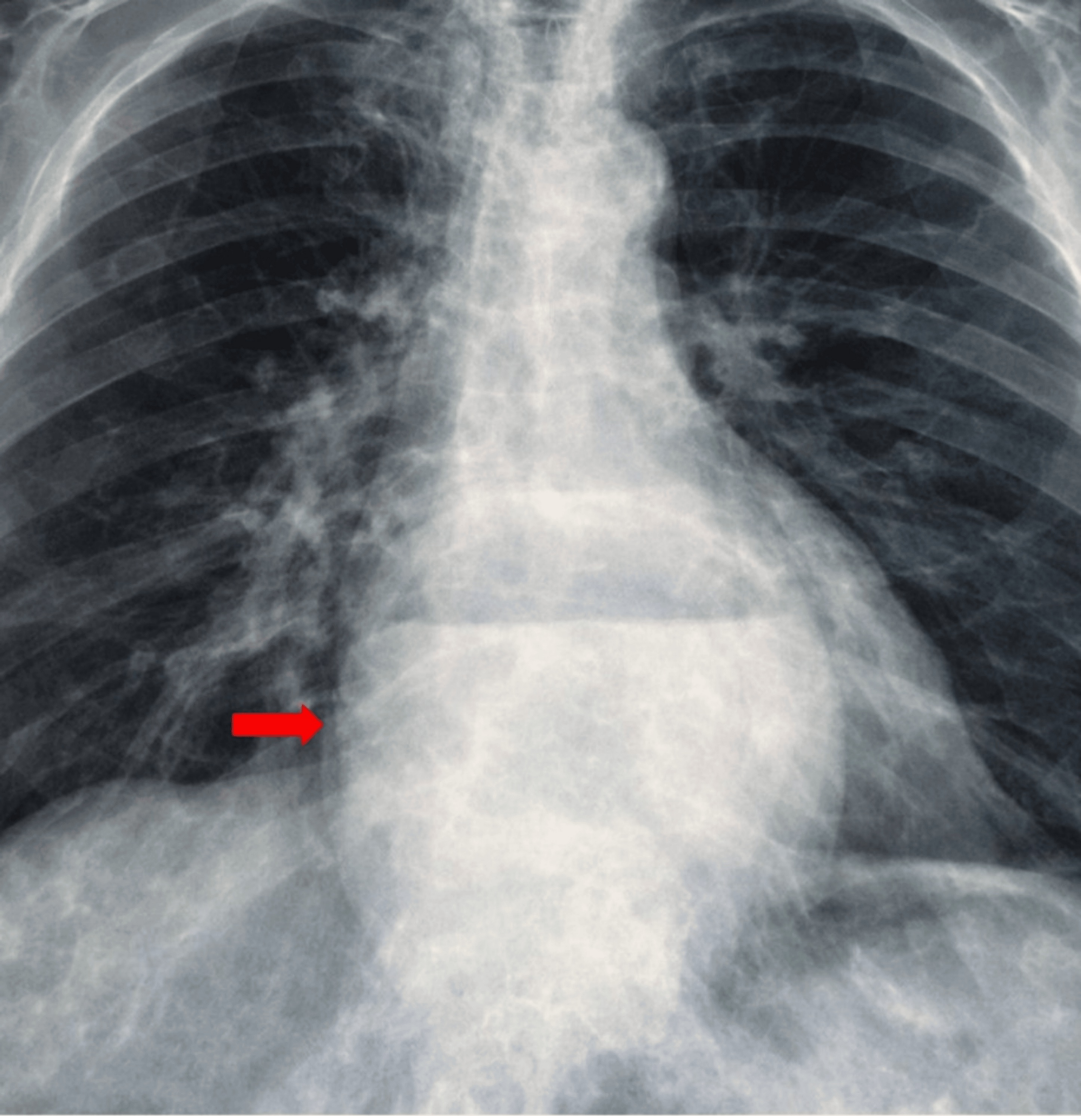
Posteroanterior chest X-ray illustrating a retrocardiac heterogeneous opacity (red arrow) with an air-fluid level mimicking a mediastinal mass

A posteroanterior chest radiograph revealed a large retrocardiac heterogeneous opacity with a distinct air-fluid level, projected over the right heart border without effacing it (negative silhouette sign), confirming its posterior location (Figure 1). At this stage, the differential diagnosis included a giant HH, mediastinal cysts, or a solid posterior mediastinal mass. Given the chronic nature of her symptoms and the striking structural abnormality on the initial radiograph, additional cardiopulmonary workups and lateral radiographs were deferred. Instead, the patient proceeded directly to a contrast-enhanced MDCT for definitive anatomical characterization.

**Figure 2:**
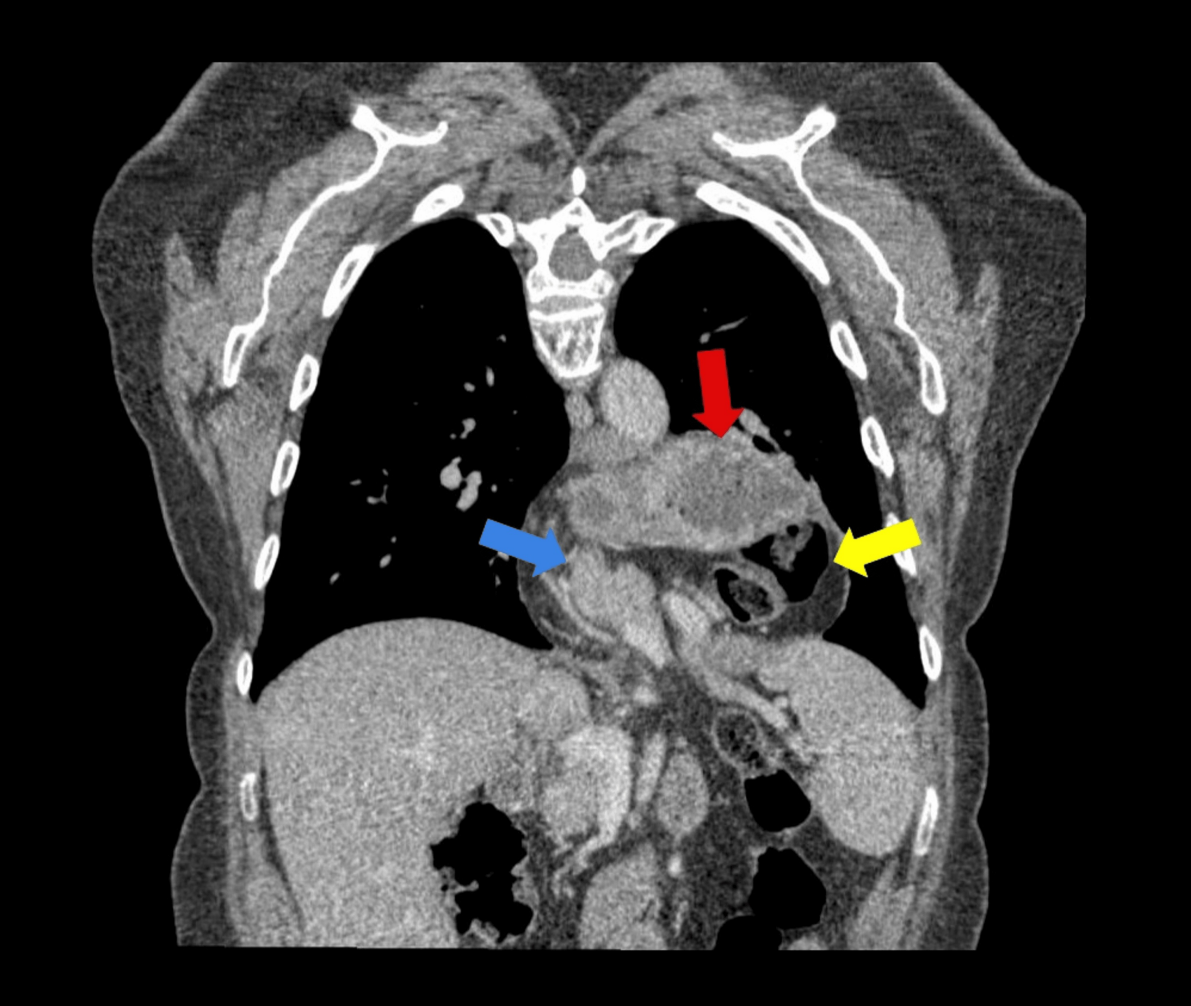
Coronal contrast-enhanced MDCT illustrating the migration of the stomach (red arrow), transverse colon (yellow arrow), and pancreas (blue arrow) into the thoracic cavity MDCT: multidetector computed tomography

The scan revealed a large esophageal hiatal defect measuring 5.5 cm in the transverse diameter, as obtained from coronal reformatted MDCT images. There was extensive herniation of abdominal contents into the posterior mediastinum, consistent with a type IV (giant) HH (Figure 2).

**Figure 3:**
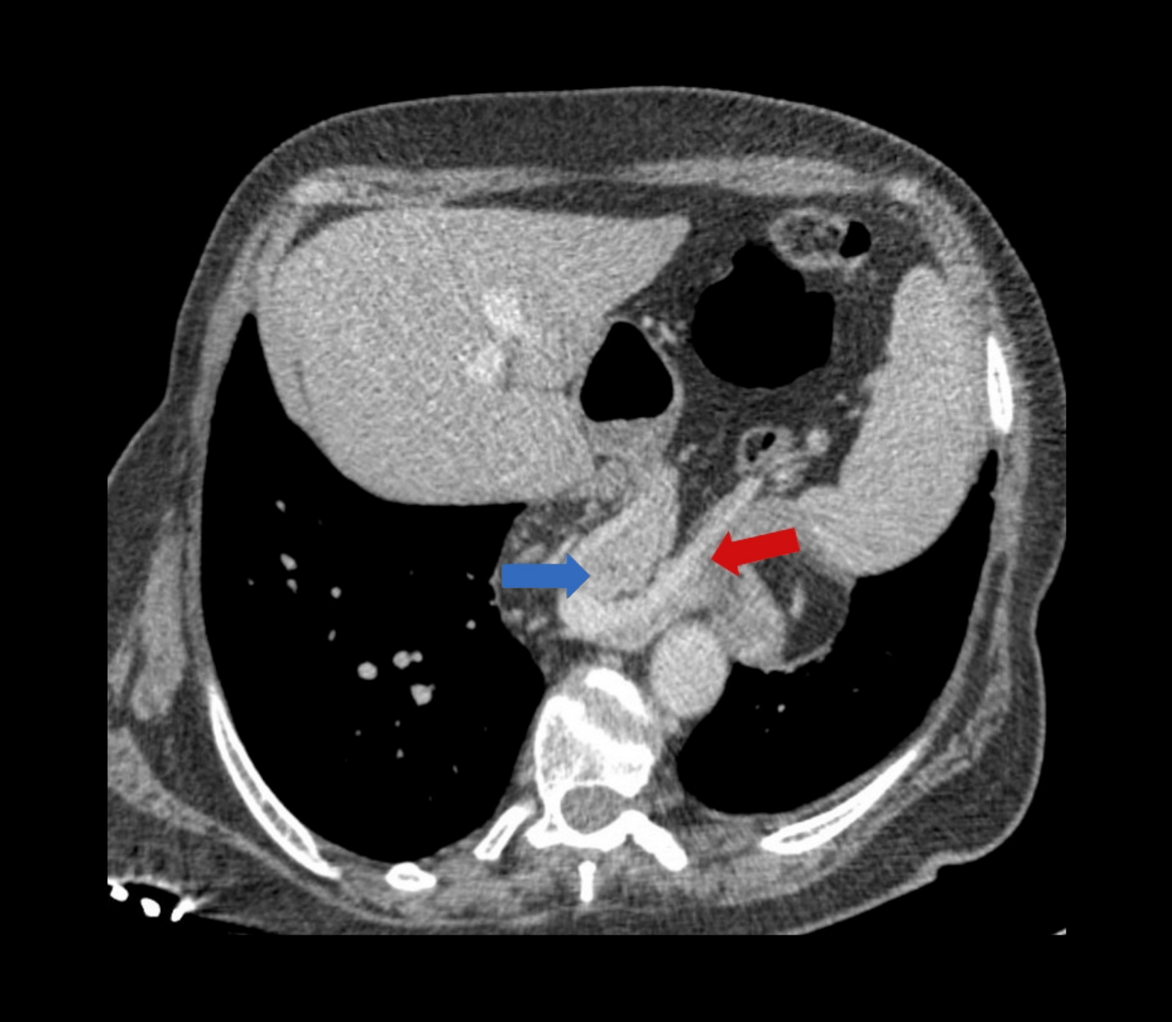
Axial contrast-enhanced MDCT illustrating the hiatal hernia sac containing the body and tail of the pancreas (blue arrow) and the splenic vessels (red arrow) MDCT: multidetector computed tomography

The hernia sac contained the stomach, a loop of the transverse colon, and mesenteric fat with several small, non-pathological, reactive lymph nodes. The GE junction was identified in an intrathoracic position. Notably, the head, body, and part of the tail of the pancreas were visualized extending superiorly through the hiatus into the left hemithorax (Figure 3).

**Figure 4:**
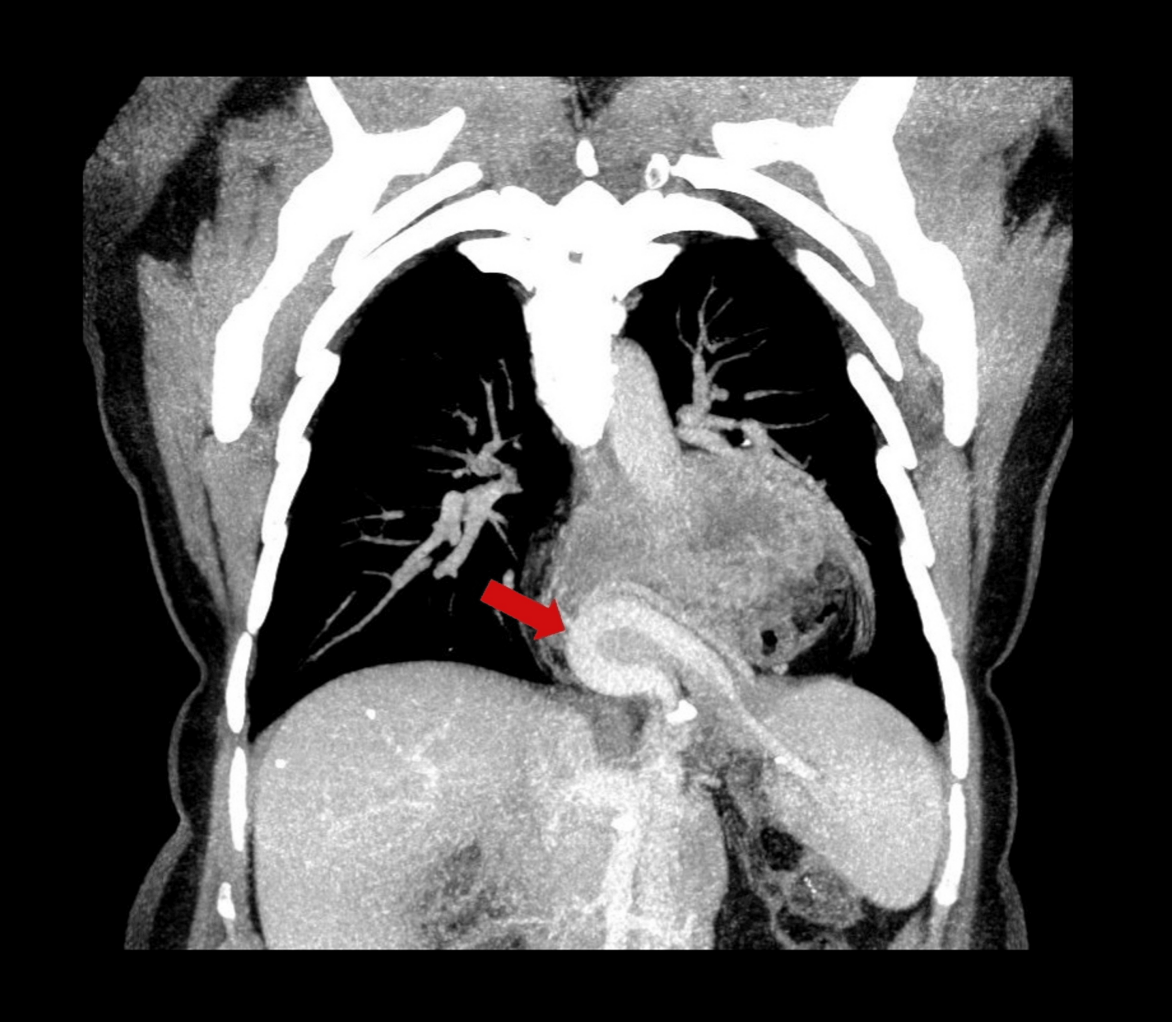
Coronal MIP reconstruction illustrating the characteristic "vascular loop" of the splenic vessels (red arrow) extending into the thorax MIP: maximum intensity projection

Vascular mapping on the CT demonstrated significant anatomical distortion. The splenic vessels were seen looping upward into the chest, following the trajectory of the pancreatic tail, before turning inferiorly to supply the spleen, which remained intra-abdominal (Figure 4). This characteristic “vascular loop” serves as a definitive diagnostic roadmap, enabling confident identification of pancreatic herniation and the exclusion of other posterior mediastinal masses, such as neurogenic tumors or lymphadenopathy. Additionally, the left gastric artery was displaced superiorly, tracking with the herniated stomach.

Crucially, the pancreatic parenchyma appeared normal in attenuation and enhancement, with no peripancreatic fat stranding, fluid collections, or ductal dilatation to suggest pancreatitis. Furthermore, there was no radiological evidence of gastric volvulus, bowel obstruction, or compromised wall enhancement in the herniated stomach or transverse colon, confirming the absence of acute incarceration. Given the diagnosis of a massive, complex HH with pancreatic herniation, the patient was referred to the department of general surgery for evaluation and potential elective repair to prevent future complications such as volvulus or ischemia.

## Discussion

Hiatal hernias are broadly classified into four types, with type IV (giant paraesophageal hernias) representing the rarest and most complex form, characterized by herniation of organs other than the stomach into the thoracic cavity [5]. Pancreatic herniation is exceptionally uncommon; a 2023 review reported only 29 cases in the literature [6]. Miyagishima et al. proposed a three-type classification system consisting of type 1 (asymptomatic), type 2 (biliary obstruction), and type 3 (acute pancreatitis) [6]. Our patient corresponds to the type 1 category, presenting with mild, nonspecific thoracic symptoms due to mass effect without biochemical evidence of pancreatitis or biliary stasis [7,8].

The extreme rarity of this condition can be attributed to the inherent stability of the pancreas. As a primarily retroperitoneal organ, the pancreas is firmly anchored to the posterior abdominal wall and the duodenum by the ligament of Treitz and the transverse mesocolon [7,9]. Herniation, therefore, requires marked laxity or failure of these fascial attachments, often exacerbated by chronically elevated intra-abdominal pressure. In our case, the herniation involved the transverse colon together with the head, body, and part of the tail of the pancreas, indicating extensive mobilization of the transverse mesocolon, a mechanism also described by Tomida et al. in cases involving the entire stomach [10].

We propose a “pulsion-traction” mechanism to explain the sequential, multi-organ migration: the initial failure of the ligament of Treitz creates a localized point of laxity, permitting the retroperitoneal pancreatic segment to herniate into the mediastinum first. This displacement then exerts secondary traction on the transverse mesocolon, effectively pulling the transverse colon and associated mesenteric structures into the thoracic cavity. This stepwise process provides a more detailed explanation for the combined herniation of intra- and retroperitoneal structures than simple global herniation models.

Contrast-enhanced MDCT remains the gold standard for diagnosis and is essential for distinguishing pancreatic herniation from mediastinal tumors. While chest radiography may reveal nonspecific findings such as retrocardiac opacities or air-fluid levels [5], it lacks precise anatomical detail. On plain radiographs, the differential diagnosis for such an opacity includes neurogenic tumors, lymphoma, aortic aneurysms, or sequestration cysts. MDCT allows definitive anatomical mapping. In our case, two key radiological signs were present: the “collar sign,” confirming the herniated nature of the mass, and the vascular anatomy. As described by Katz et al., the splenic vessels accompany the pancreatic tail, forming a characteristic “vascular loop” that extends into the chest and returns to the abdomen [7]. This loop differentiates the condition from Bochdalek hernias, where the spleen itself often herniates, creating a continuous cephalad vascular trajectory. Recognition of this loop, together with the displaced left gastric artery, allows a confident diagnosis and effectively excludes neoplastic etiologies.

Management depends on the clinical presentation and the patient’s comorbidities. While types 2 and 3 require urgent intervention [4,6,11], the optimal management of type 1 patients remains debated. Forrest et al. recently reported an 84-year-old female managed conservatively due to high surgical risk [12]. However, in younger patients with significant life expectancy, the risk of complications, such as ischemia from vascular traction, volvulus, or incarceration, supports surgical repair [13,14]. Nasri et al. highlight that even conservative management demands careful and ongoing monitoring [9]. Given our patient’s age (60 years), chronic symptoms, and the vascular complexity of the hernia, we elected for a multidisciplinary surgical referral to prevent catastrophic incarceration.

A major limitation of this report is the lack of operative confirmation, as the anatomical findings described are based solely on MDCT interpretation, and surgical exploration has not yet been performed. Nevertheless, the primary focus of this report is to emphasize the diagnostic value of MDCT in characterizing this rare anatomical variant.

## Conclusions

Partial intrathoracic migration of the pancreas is a rare but critical complication of a giant HH that can mimic a mediastinal mass on plain radiography. Radiologists should maintain a high index of suspicion for this diagnosis in patients with giant (type IV) HH, specifically looking for the characteristic looping of the splenic vessels on MDCT. Accurate preoperative identification of the pancreas and its associated vasculature is vital to guide surgical planning, specifically in selecting the optimal approach and preventing iatrogenic injury to the splenic vessels or pancreatic parenchyma during sac dissection.
